# Trace Metals, Crude Protein, and TGA-FTIR Analysis of Evolved Gas Products in the Thermal Decomposition of Roasted *Mopane* Worms, Sweet Corn, and Peanuts

**DOI:** 10.1155/2022/1509569

**Published:** 2022-10-25

**Authors:** Nonkululeko S. Masite, Somandla Ncube, Lawrence M. Madikizela, Fanyana M. Mtunzi, Vusumzi E. Pakade

**Affiliations:** ^1^Department of Chemistry, Private Bag X 021, Vaal University of Technology, Vanderbijlpark, South Africa; ^2^Department of Chemistry, Sefako Makgatho Health Sciences University, P.O Box 60, Medunsa 0204, South Africa; ^3^Institute for Nanotechnology and Water Sustainability, College of Science, Engineering and Technology, University of South Africa, Florida Science Campus, 1710, South Africa

## Abstract

The thermal behavior of *mopane* worms (*Imbrasia belina*), roasted peanuts (*Arachis hypogaea* L.), and sweet corn (*Zea mays* L. saccharata) was investigated under inert conditions using the TGA-FTIR analytical technique heated from 64 to 844°C at a heating rate of 20°C/min. The degradation patterns of the food samples differed as sweet corn and peanuts exhibited four degradation stages 188, 248, 315, and 432°C and 145, 249, 322, and 435°C, respectively. *Mopane* worms displayed three (106, 398, and 403°C). The different decomposition patterns together with the types of evolved gases shown by FTIR analysis justified the varied biochemical and chemical composition of foods. The common evolved gas species between the food samples were H_2_O, CO_2_, P=O, CO, and CH_4_ but *mopane* worms showed two extra different bands of C-N and N-H. Higher volumes of evolved gases were recorded at temperatures between 276 and 450°C, which are higher than the usual cooking temperature of 150°C. This means that the food maintained its nutritional value at the cooking temperature. *Mopane* worms were found to contain twice and four times crude protein content than peanuts and corn, respectively. Only total arsenic metal was reported to be above threshold limits.

## 1. Introduction

According to Ellis et al. [1], the processing of food is an intricate process that needs to be monitored promptly and remedies any quality degenerating concerns as soon as they occur [1]. Thermogravimetric analysis Fourier transform infrared spectroscopy (TGA-FTIR) is a versatile analytical technique used for the simultaneous and continuous real-time analysis of volatile gaseous products from complex samples [2–4]. The technique has largely been applied in the analysis of evolved gases from food processing wastes such as grape seeds, cherry stones, chestnut shells, pork, and rice [5, 6]. The thermal decomposition analysis of inulin carbohydrates by TGA-DSC-FTIR confirmed the evolution of CO_2_ and CO gases at about 214°C [7]. Recently, the evolved gas analysis mass spectrometry was used for the determination of nonvolatile and volatile compounds from various spices [8]. Few studies have reported the application of TGA-FTIR analysis of evolved gas products from food components [9–11].

*Mopane* worms are classified as nontimber forest products (NTFPs) harvested by residents of Limpopo Province (South Africa) for consumption and sold to generate household income [12, 13]. In 2009, the income per annum generated by *mopane* worm traders in the Limpopo Province, South Africa, was estimated to be around R20,000 (US$2457) [14, 15]. The interesting foot about these caterpillars is that they feed almost exclusively on *Mopane* tree (*Colophospermum mopane*) [13], thus minimizing the impact of food poisoning but the processing could be a source of contamination. Like other edible insects of South Africa, *mopane* worms are a good source of nutrients such as proteins and vitamins [16, 17]. A literature has reported the crude protein content of *mopane* worms ranging between 58 and 70% [18, 19] further showing the nutritional value of these insects. In contrast, sweet corn contains high starch content (72.3%), low sugar content (2.3%), and moderate protein content (11.2%) [20]. As a member of the legume family, peanuts contain approximately 23% of carbohydrates, 48% of oil, and 26% of protein [21].

In South Africa, it is common to find street vendors selling roasted *mopane* worms, sweet corn, and peanuts particularly during their harvest seasons. The foods are prepared along the streets using open heat sources from wood or charcoal. The foods are exposed directly to open heat until they are matured to eat. Thus, this study is aimed at understanding the changes in food constituents such as gas evolving patterns during heat treatment. The chemical changes occurring during the preparation and processing of food at high temperatures influence many properties of the food, including taste and sensory [22]. Furthermore, it was essential to study the thermal decomposition of *mopane* worms, a South African indigenous food, in relation to various internationally known foods. Hence, the objective of the present work was to study the thermal decomposition of roasted *mopane* worms (*Imbrasia belina*), peanuts (*Arachis hypogaea*), and sweet corn (*Zea mays* L. saccharata). The roasted foods were subjected to a TGA-FTIR instrumentation for the analysis decomposition patterns and evolved gases during the thermal treatment in order to obtain the characteristic chemical fingerprint associated with each food type. Also, the crude protein and toxic trace metal contents of *mopane* worms were monitored and compared with those of roasted peanuts and sweet corn to gain insights on their nutritional value and potential toxicities. Such information is crucial for the consumer to spring awareness regarding the presence of any suspected dangers of toxins in foods. To the best of our knowledge, no such study has been done in the past.

## 2. Experimental

### 2.1. Chemicals and Materials

Reagent grade chemicals such as hydrochloric acid (37%), sulfuric acid (98%), nitric acid (70%), and nitrate salts of As, Cd, Co, Cr, Ni, and Pb, purchased from Merck Chemical Co. (Johannesburg, South Africa), were used as received. *Mopane* worms, sweet corn, and peanuts were sourced from Tshakhuma Fresh Produce Market and Makhado Fruit Market located in Limpopo Province, South Africa, around the beginning of 2019 year. The samples were roasted as described in literature [23, 24] in an oven vessel for 30 min at 150°C. The samples were roasted from raw state to edible state in one cycle of heating. Upon cooling to room temperature, the samples were crushed and ground into fine powder using a pestle and mortar. The powdered samples were freeze-dried with ALPHA 1–2 plus freeze drier from Lasec (Cape Town, South Africa) and stored in air-tight bottles in the refrigerator (-20°C) when not in use.

### 2.2. Crude Protein Analysis of *Mopane* Worms, Peanut, and Corn Food Samples

The protein composition analysis of *mopane* worms, peanut, and corn food samples was carried out using the Kjeldahl method [25]. The samples were analyzed individually and in triplicate. As a statistical measure, the relative standard deviation (RSD), which represents the precision, was computed from the ratio of standard deviation to the mean of three replicates. Briefly, about 0.20 g of the ground sample was weighed and transferred to a Kjeldahl flask. Two Kjeldahl tablets were added to the flask followed by the addition of 20 mL of sulfuric acid. The sample was then heated progressively in the speed digester until a slightly yellowish or green color emerged (depending on the used Kjeldahl tablets). The sample was allowed to cool to room temperature. The distillation unit was switched on and primed prior to sample distillation. The sample was then distilled for 5 min, followed by titration with 0.1 M hydrochloric acid to a faint pink color. Thereafter, the crude protein content was calculated according to

(1) $$\begin{array}{c} \text{Crude protein content}=\frac{T\times M\times Mw\times 6.25}{\left(W_{t}/V\right)}, \end{array}$$

where *T* is the titration value, *M* is the molarity of HCl, *W*_t_ is the weight of sample (±0.20 g), V is the volume of solution (20 mL), 6.25 is the nitrogen-to-protein conversion factor, and Mw is the molar mass of nitrogen in g·mol^−1^.

### 2.3. Analysis of *Mopane* Worms, Peanut, and Corn Food Samples by TGA-FTIR

Thermogravimetric analysis (TL9000, PerkinElmer) was conducted in an inert (nitrogen-N_2_) environment at a rate of 20°C min^−1^ from 64-844°C. About 20 mg of each sample was tested. The TGA test was done once per each sample. The temperature of the transition line (TL9000, PerkinElmer) between TGA and FTIR was maintained at 250°C to avoid the condensation of the evolved volatiles. The FTIR was set to remove H_2_O from the results; thus, no H_2_O profile has been observed.

### 2.4. Trace Metal Analysis and Health Risk Assessment

Trace metal analysis was done on the three food samples to conduct a health risk assessment due to six selected heavy metals with potential health effects on the human body. Triplicate sample treatment and analyses were performed for each sample. The dry samples were digested using a BIOBASE MLS-1200 MEG multifunctional bolt design microwave digester (BIOBASE, Qingdao, China). The procedure involved weighing 2.5 g of the dry powdered samples into microwave extraction bomb vials followed by the addition of 12 mL of an acid mixture (9 mL HNO_3_+3 mL HCl). Digestion was executed according to a previously reported method in which the digester temperature was set at 175°C for 45 min [26]. The digestate was further diluted to 30 mL using deionized water, filtered through 0.2 *μ*m syringe filters, and finally analyzed for the targeted trace metal content using an inductively coupled plasma optical emission spectrometer (ICP-OES) from PerkinElmer (South Africa). Where applicable, RSD values were determined from the ratio of standard deviation to the mean of three replicates.

The measured concentrations of the metals using ICP-OES were used in the calculation of risk assessment parameters as given by Equations (2)–(4). For those heavy metals with known cancer risk, their health risk due to consumption of the *mopane* worms, peanuts, and sweet corn was estimated as a target cancer risk (TCR) value using Equation (4). The ingestion rate (IR) for each food source was estimated at 0.00395, 0.0283, and 0.245 kg day^−1^·dw for *mopane* worms, peanuts, and sweet corn, respectively. Based on anecdotal evidence, the *mopane* worms are consumed at 3–5 times per week over 2 months, which equates to 1.44 kg per year (0.00395 kg day^−1^, assuming each worm weighs 12 g and each meal consists of 5 worms) (http://Mopaneworms.com/ accessed 27/01/2021). As of 2017/8, sweet corn consumption in South Africa is estimated at 5.2 million tons, which equates to 0.245 kg day^−1^ per person (https://www.statista.com/statistics/1135184/consumption-of-processed-maize-in-south-africa/ accessed 27/01/2021). For peanuts, the estimated IR was based on health recommendations of 1 ounce (0.0283 kg per day) (https://peanut-institute.com/ accessed 27/01/2021). However, higher peanut consumption is advised to control some health conditions. For example, peanuts may control diabetes when consumed at ≥140 g per day [27].

(2) $$\begin{array}{c} \text{ EDI}=\frac{C_{m}\times \text{EF}\times \text{ED}\times \text{IR}}{\text{BW}\times \text{AT}}, \end{array}$$

where EDI is the estimated daily intake (mg kg^−1^ day^−1^), *C*_*m*_ is the mean concentration of the element in the food source (mg kg^−1^·dw), EF is exposure frequency (365 days·yr^−1^), ED is exposure duration (70 years), IR is the ingestion rate per person (0.00395, 0.0283, and 0.245 kg day^−1^·dw for *mopane* worms, peanuts, and sweet corn, respectively), BW is the bodyweight of an adult (70 kg in South Africa), and AT is the average time (365 days/year × 70 years = 25550 days).

(3) $$\begin{array}{c} \text{ THQ}=\frac{\text{EDI}}{{\text{Rf}}_{D}}, \end{array}$$

where THQ is the target hazard quotient and Rf_*D*_ is the reference daily oral intake (mg kg^−1^ day^−1^) of the element according to WHO/FAO.

(4) $$\begin{array}{c} \text{TCR}=\frac{\text{EF}\times \text{ED}\times \text{FIR}\times {\text{C}}_{\text{m}}\times \text{CSF}}{\left(\text{BW}\times \text{AT}\right)}, \end{array}$$

where TCR is the target cancer risk due to a carcinogenic element and CSF is the cancer slope factor of the carcinogenic element (kg-day·mg^−1^).

## 3. Results and Discussion

### 3.1. Crude Protein Analysis

Results of the investigated *mopane* worms, peanut, and sweet corn crude protein content are shown in Table 1. The crude protein content in *mopane* worms was 60.5%. These results corresponded well with those reported in the literature which indicated that *mopane* worms contain about 58% [18] and 70% [19] of crude protein content. According to Potgieter et al. [28], by unit weight, dried *mopane* worms have thrice the protein content of beef and the nutritional value exceeds that of milk, beef, and chicken [28]. Elsewhere, Tang et al. noted that although the amino acid compositions of traditional meat and edible insects are mostly similar, edible insects typically contain more crude protein than traditional meat [29]. Hundred grams of dried *mopane* worms are said to provide up to 76% of daily protein need by a human.

The crude protein for peanuts was found to be 32.47%, and these results correspond to those reported by Atasie et al. for groundnut which indicated 38.61% crude protein [30]. Animal-based foods provide all of the protein required by the human body, meaning they have all of the essential amino acids. Like meat, peanuts have been said to possess the essential amino acids that are needed for the synthesis of protein, making them an important part of the human diet. Due to their high plant protein content compared to other nuts and their low cost, peanuts are also sometimes referred to as a poverty-stricken man's protein.

The crude protein content in sweet corn was found to be 17.09%. The obtained crude protein values are not far from those reported by Budak and Aydemir [20] which indicated 11.2% for the Sakarya composite type of sweet corn as well as those reported by Nwalo which indicated 8.7% for sweet corn [31].

### 3.2. Characterization and Compositional Analysis of *Mopane* Worms, Peanut, and Corn Food Samples

#### 3.2.1. FTIR Analysis of Evolved Gas Products

The hyphenation of TGA with FTIR allowed for the simultaneous determination of sample thermal stability, decomposition patterns, and functional groups of the evolved gas products. The commonality between the food samples under study is that they can be processed for consumption by exposure to open heat sources such as charcoal (with an estimated low temperature of about 200-300°C [32]). During food processing at high temperatures, there is a likelihood of food contamination through the formation of various compounds [33] and a possibility of changing the nutritional value of unprocessed food [34]. Since roasting can be performed at high temperatures, the loss of certain components of volatile compounds is expected. Based on this background, FTIR was used to monitor the decomposition patterns and allowed for the analysis of the chemical fingerprint of the evolved gas products of South African-based traditional foods after heat processing.

**Figure 1:**
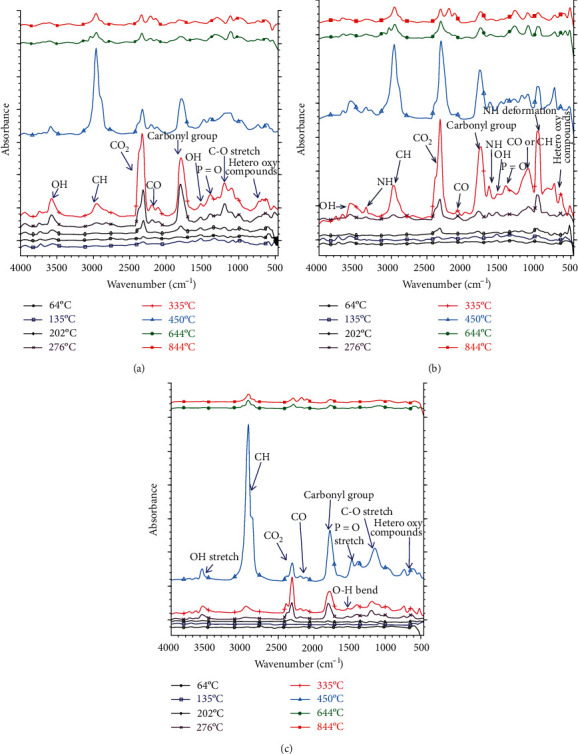
FTIR analysis of (a) sweet corn, (b) *mopane* worm, and (c) peanuts with an increase in temperature from 64 to 844°C.

Figures 1(a)–1(c) show the FTIR spectra of roasted sweet corn, *mopane* worms, and peanuts with increasing temperatures from 64 to 844°C. The spectra show that there was an evolution of degradation products from 64 to 202°C. At a temperature of 202°C, medium, sharp stretching vibration characteristic of free OH hydroxyl from alcohols (3450-4000 cm^−1^), O=C=O stretch of CO_2_ (2250-2396 cm^−1^), hydroxyl C-OH bending due to phenolic groups and/or light aromatics (1300-1590 cm^−1^) [35], and carboxyl C=O groups (1707-1856 cm^−1^) were observed for sweet corn probably due to the high starch content (Figure 1(a)). The possible reactions for the evolution of the mentioned gases and functional groups could include the decomposition of the amylose and amylopectin polysaccharides coupled with depolymerization and elimination of polyhydroxyl groups [36]. For *mopane* worms, only the O=C=O stretch of CO_2_ (2250-2396 cm^−1^) and N-H stretching (962 cm^−1^) for -NH_2_/-NH groups were observed at a temperature of 202°C (Figure 1(b)). Similarly, for peanuts, only the O=C=O stretch of CO_2_ (2250-2396 cm^−1^) peak was observed at 202°C (Figure 1(c)). The differences in gas evolution profiles are related to the chemical make-up of each food sample. Food samples are known to contain, among other constituents, macromolecular polymers such as fats, starch, and proteins [5]. The deamination and decarboxylation of amino acids during heat treatment of food is associated with the evolution of CO_2_ and NH_3_ gases as depicted in Equations (5) and (6) [11, 37]. Thus, among the different foods, one can posit that *mopane* worms contain more amino acid proteins than corn and peanuts due to the evolution of N-H bands as well as the intensity of the peaks.

(5) $$\begin{array}{c} {\text{H}}_{2}\text{NC}\left(\text{R}\right)\text{COOH }\overset{∆}{⟶}\;{\text{RCH}}_{2}{\text{NH}}_{2}+{\text{CO}}_{2\;\left(g\right)}, \end{array}$$

(6) $$\begin{array}{c} {\text{H}}_{2}\text{NC}\left(\text{R}\right)\text{COOH }\overset{∆}{⟶}\text{ RC}\left(\text{O}\right)\text{COOH}+{\text{NH}}_{3\;\left(g\right)}. \end{array}$$

With increased temperature from 202 to 276°C, the appearance of additional contributions related to stretching vibrations of C-O and C-H in the regions of 2025 to 2240 cm^−1^ and 2620 to 3035 cm^−1^, respectively; CO stretch and hetero-oxy compounds in the regions of 1456 and 1596 cm^−1^ and 400-800cm^−1^, respectively, are noticed for sweet corn. For *mopane* worms (Figure 1(b)), the main groups identified in the temperature range of 202 to 276°C were the emergence of additional contributions to N-H peak (3332 cm^−1^), aliphatic C-H vibrations (2620-3035 cm^−1^), C-O peaks (2025-2240 cm^−1^), a strong C=O stretch of carboxylic acid monomers (1765 cm^−1^), N-H bending (1625 cm^−1^), in-plane O-H bending of alcohols and phenols (1509 cm^−1^), N-H stretching deformation (962 and 930 cm^−1^), and possibly the hetero-oxy compounds (400–800cm^−1^). Peanuts exhibited an bonded hydroxyl (O-H) peak (3561 cm^−1^), C-H vibrations (3023–2777 cm^−1^), O=C=O stretch of CO_2_ (2244–2412 cm^−1^), stretching vibrations of C-O (2047–2215 cm^−1^), a strong C=O stretch of carboxylic acid monomers (1765 cm^−1^), in-plane O-H bending of alcohols and phenols (1510 cm^−1^), P=O stretch (1340–1464 cm^−1^), C-OH stretch (1040–1159 cm^−1^), and hetero-oxy compounds (400–800 cm^−1^) at 202 to 276°C. A further increase in temperature from 276 to 450°C yielded no change in the characteristic peaks of sweet corn and peanuts but only noticed an increase in intensity and absence of the O-H bending (1512 and 1510 cm^−1^). In addition, due to the increase in intensity, there was a development of a broad O-H stretch due to alcohols and phenols (3568 cm^−1^), a strong N-O stretch of nitro compounds (1535 and 1510 cm^−1^), P=O stretch (1340–1464 cm^−1^), and C-O or C-N stretch (1040–1159 cm^−1^) in *mopane* worms between 276 and 450°C. At 644–844°C, the intensity of all peaks in the samples was significantly reduced. There was a notable presence of C-H, CO_2_, and C-O stretching vibrations and carbonyl in peanut and C-H stretching, CO stretching vibration, carbonyl groups, P=O stretch, C-O or C-N, and N-H symmetric deformation in *mopane* worms. The high crude protein content reported in Table 1 is linked to the observed C-O, C-N, and N-H deformations in FTIR of *mopane* worms. Unlike in *mopane* worms, there was no evolution of nitrogen containing compounds from the sweet corn and peanuts samples even though the crude protein analysis showed possible 17 and 32% of protein in these foods, respectively. This could be due to the overriding effect of the high starch content on these samples.

Based on these observations, the following deductions regarding the nutritional value of the food samples and their significance during roasting were made: (i) the chemical fingerprinting of evolved gas products from different foods shared some common features in terms of generation of O-H, P=O, CO_2_, CO, and CH peaks even though the intensity varied but only *mopane* worms exhibited N-H and C-N peaks; (ii) the presence of N-H, C-N, C-O, and ammonia groups in *mopane* worms were probably an indication of the existence of volatile organic compounds such as amines, amides, hydrocarbons, and possibly amino acids [38]; (iii) the P=O could be an indication of the presence of phosphates nutrients in the food samples; (iv) the OH groups could be originating from phenols and alcohol groups of the protein, fat, and starch [39]; and (v) the presence of a strong C-H peak at 450°C in all samples could be attributed to evolution of CH_4_ gas from the food samples which could be a result of cleavage and rearrangement of aliphatic hydrocarbons, aromatic rings opening, and methanation [5].

The functional groups observed after thermally treating the different food samples are summarized in Table 2. It is evident that the effect of heat treatment led to the formation/evolution of different functional groups owing to the intrinsic chemical make-up of the raw material. The table also shows at which range of temperatures were certain functional groups observed. For instance, the roasted sweet corn and peanut samples exhibited O-H, O=C=O asymmetric stretching, and carbonyl C=O stretching vibrations at temperatures ranging from 64 to 200°C (Tables 2(a) and 2(c)). In contrast, nothing happened at these temperatures for *mopane* worms (Table 2(b)). The results confirmed that the food samples possess differences in their chemical make-up. Probably, the reason why the O-H and C-O peaks were not observed in *mopane* worm samples was because of the absence of sugars in *mopane* worms which make them thermally stable to about 200°C. A study attributed the presence of carbonyls, C-O-C, and O-H bands at low temperatures to the breakdown of the single sugar ring [40].

#### 3.2.2. Thermal Analysis of *Mopane* Worms, Peanut, and Sweet Corn

**Figure 2:**
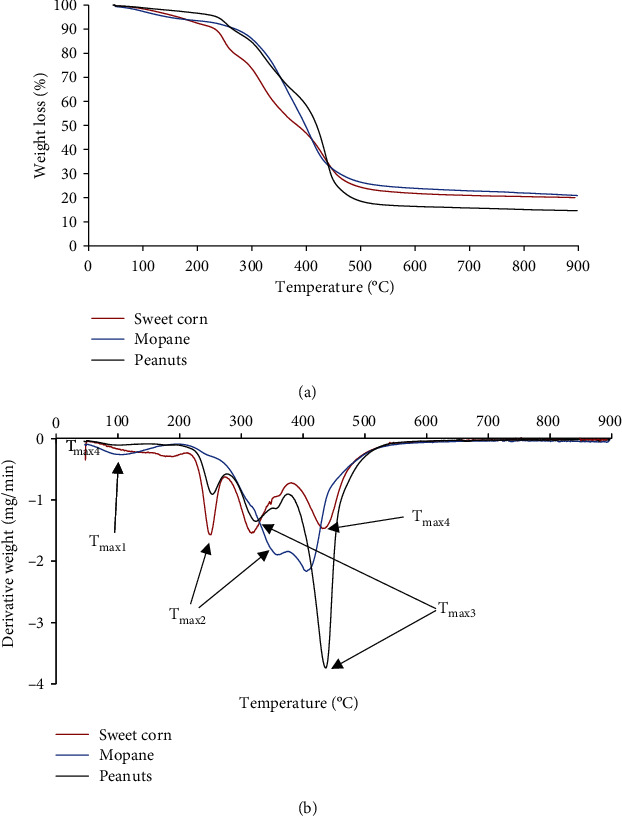
TGA and dTGA curves for sweet corn, *mopane* worms, and peanuts.

The study of thermal behaviors of foods is critical for understanding the decomposition patterns and optimizing the processing conditions.  Figure 2(a) depicts the TGA curves for the roasted food samples while Figure 2(b) shows the derivative curves. The TGA curves in Figure 2(a) show that all food samples exhibit multiple degradation stages and that *mopane* worms had the highest residue of about 22%. The multiple degradation stages can be further observed in Figure 2(b) where the exact temperatures of maximum degradation are shown. It can be further observed in Figure 2(b) that sweet corn and peanuts share a similar decomposition profile consisting of four peaks. For sweet corn, the four degradation stages were at 188, 248, 315, and 432°C, while for peanuts, the temperatures of decomposition were at 145, 249, 322, and 435°C. *Mopane* worms displayed three degradation stages at 106, 398, and 403°C. Only the *mopane* worm sample exhibited a degradation peak which can be attributed to the loss of moisture at 106°C. Elsewhere [41], it was reported that food samples roasted at high temperature may still absorb moisture from storage and weighing stages. Peanuts and corn exhibited no peaks attributed to loss of moisture, which may indicate that they are not as water receptive as the *mopane* samples. For the sweet corn sample, the thermal events taking place at 188°C could be evolution of light volatiles resulting from decomposition of sugars and carbohydrates [40, 42], while those at 249, 315, and 432°C were ascribed to onset pyrolysis accompanied by the decomposition of starch, proteins, and/or lipids [41, 42]. The same can be said of peanut degradations at 249, 322, and 435°C. Among its other ingredients, peanuts contain dietary fiber. The decomposition temperature for fibers was reported to range between 210 and 310°C [43]. It has also been noted elsewhere that the pyrolysis of starch at about 350°C may lead to the release of carbon monoxide, carbon dioxide, water, and acetaldehyde [44]. In comparison to the other two foods, *mopane* worms are insects while the other two are originating from plants. Hence, *mopane* worms do not possess starch and lignin decomposition temperatures but total decomposition of the insects at 398 and 403°C probably due to breakdown of lipids [42].

Table 3 summarizes the calculated data from the TGA curves. If the *T*_max1_ at 106°C for *mopane* worms is attributed to loss of moisture, then it makes the *mopane* worms to exhibit the highest thermal stability among the three food samples because its other degradation peak is observed at 398°C and this happens to be the *T*_50%_ (50% decomposition of the sample), while the last event for *mopane* worms is at 403°C. Peanuts display the highest onset degradation temperature of 236°C, and 50% degradation is observed at 419°C but there are other major events happening at 249 and 322°C. Sweet corn had an onset degradation temperature of 165°C, and 50% degradation is observed at 384°C but with other major events taking place at 188, 248, and 315°C. These results reveal that *mopane* worms exhibited the highest thermal stability in comparison to the other food samples because the major thermal event only took place at 398°C. These results are collaborated by the observations made in Table 2(b) where there were no gas evolutions for *mopane* up to 200°C of heating. Sweet corns exhibited the lowest thermal stability among the tested samples probably due to its high sugar content.

The char residue for all samples is also presented in Table 3. Comparison of the percentage of char yield was fixed at 800°C. The presented data clearly shows that all samples formed char, and *mopane* worms have the highest char residue of 21.94%, followed by sweet corn at 20.46%, and peanuts with a char yield of 15.08%. In thermal analysis, char content is related to the presence of inorganic material; thus, the percent char content can be attributed to the presence of minerals in food samples with *mopane* worms exhibiting the highest content than other foods. As per FTIR analysis, the OH, CO_2_, and C-H functional groups were observed between temperatures 202 and 450°C, and the major degradation in TGA occurred between 200 and 500°C for all samples. Thus, the presence of O-H could signify the evolution of water and the decomposition of starch, phenol, and alcohol groups from the food samples [40]. Specifically for *mopane* worms, protein is a primary component; therefore, N-H, O-H, and C-O functional groups arise from carboxyl group dehydration of amino acids [5].

#### 3.2.3. Evolution Profiles

**Figure 3:**
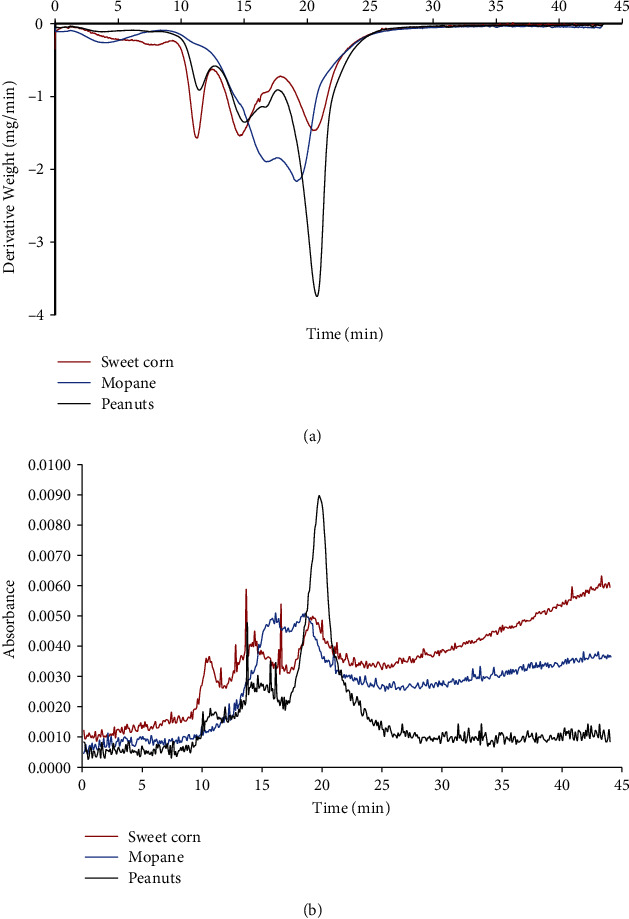
(a) dTGA curves and (b) evolution profile of the degradation products of sweet corn, *mopane* worm, and peanut samples.

Figure 3 exhibits the dTGA curves and the evolution profile of the degradation products of the samples. It could be observed that all food samples were completely degraded within 25 min and a maximum temperature of 500°C was reached. The evolution peaks in Figure 3(b) clearly show that the food samples were constituted by different ingredients. These could be related to the nutritional component of food.

### 3.3. Trace Metal Analysis

The occurrence of various metals which included As, Cd, Co, Ni, and Pb in food samples was investigated.  Table 4 shows that only As was detected in all three food samples with a concentration range of 18.2-74.6 mg kg^−1^ with the highest concentration recorded for sweet corn. The method detection limits for As, Cd, Co, Cr, Ni, and Pb were 4.97, 1.39, 5.02, 1.57, 8.09, and 14.1 *μ*g kg^−1^, respectively. The recorded As concentrations were all above the recommended WHO guideline limits in food sources set at 0.5 mg kg^−1^. However, *mopane* worms are seasonal and their potential impact on increasing As accumulation in the human body is very low as presented by low EDI, HQ, and TCR values (Table 4). For peanuts and sweet corn, the HQ and TCR were all higher than noneffect. Most importantly, the potential health effects were more pronounced for sweet corn with its HQ and CSR values as high as 86.9 and 0.391, respectively. For HQ > 1 and TCR > 0.1, consumption of the food source is considered unsafe and the risk of developing cancerous ailments is high [26]. Elsewhere, As has also been detected in lower concentrations of up to 0.0169 mg kg^−1^ in Brazilian peanuts [45]. In China, Wong et al. [46] reported 0.006 mg kg^−1^ and 0.0015 mg kg^−1^ in peanuts and corn, respectively [46]. On the other hand, other heavy metals (Cd, Cr, Ni, and Pb) have been detected in peanuts from Turkey [21], southern China [47], and Nigeria [48]. The concentration of toxic metals in the plant leaves on which *mopane* worms were extracted from was compared to those obtained from *mopane* worms, and substantial bioaccumulation of Cd, Cu, Co, and Mn in *mopane* worms was reported. The samples were collected from the Kruger National Park in the Limpopo Province of South Africa, and the highest concentrations of Cd, Cu, Co, and Mn were 4.02, 47.73, 4.52, and 3.37 *μ*g g^−1^, respectively, and all these values were above the recommended guidelines by the European Union [49]. However, in the current study, the concentrations of metals were well below detection limits and we can conclude that the consumption of *mopane* worms remains safe from potential metal contamination, while concentrations of As in sweet corn should be of serious concern. Water has been reported to be one of the major contributors of inorganic arsenic in food [46]. Thus, contamination of food crops during irrigation is a possibility for the high As concentration in sweet corn. More studies are needed to ascertain the source of As in these three food sources. For sweet corn, a more detailed study and risk assessment with samples from across the country is needed.

## 4. Conclusions

The analysis of evolved gases by FTIR and degradation patterns by TGA revealed that *mopane* worms, peanuts, and sweet corn food samples significantly decomposed between temperatures of 200 and 500°C. Under the inert conditions used during TGA analysis, the food decompositions were less pronounced under 150°C implying that the foods maintained their biochemical and chemical characteristics up to slightly above 150°C. In fact, the *mopane* worms exhibited the highest thermal stability as there was no evolution of gases up to 200°C of heat, and the major thermal event took place at 398°C. Sweet corn on the other hand displayed the lowest thermal stability with major thermal events taking place at 188, 248, and 315°C. The decomposition patterns of sweet corn and peanuts almost overlapped and were distinct when compared to *mopane* worm decomposition patterns. The FTIR analysis showed that the food samples exhibited similar chemical fingerprint characteristics of the evolved gases in terms of generation of O-H, P=O, CO_2_, CO, and CH peaks. However, *mopane* worms also exhibited N-H and C-N peaks which were attributed to the degradation of amino acid and protein. The manifestation of quantitatively larger volumes of gas products by *mopane* worms at 276-450°C could be linked to the presence of high protein content. *Mopane* worms have the highest protein content (60.5%), in comparison to the other food samples studied, peanuts (32%) and sweet corn (17.8%). Among the heavy metals analyzed, only inorganic arsenic was found to be higher than the threshold limit in all three food samples. Overall, this study provided crucial information on the nutritional value and potential toxicity of heat-processed *mopane* worms, peanuts, and sweet corn.

## Figures and Tables

**Table 1:** Percentage mean crude protein (*n* = 3) of *mopane* worms, sweet corn, and peanuts.

Sample	Crude protein% ± RSD (%)
*Mopane* worms	60.50 ± 2.74
Peanuts	32.47 ± 1.95
Sweet corn	17.09 ± 2.02

**Table:** (a) Summary of functional groups in sweet corn

Temperature (°C)	O-H stretching	C-H asymmetric stretch	Asymmetrical stretching in O=C=O	Stretching vibration in C-O	Carbonyl group	O-H bending	C-O stretch	Hetero-oxy compounds
64-200°C	√		√		√	√		
200-300°C	√	√	√	√	√	√	√	√
300-450°C	√	√	√	√	√	√	√	√
>450°C		√	√	√	√		√	

**Table:** (b) *Mopane* worm function group summary

Temperature (°C)	O-H stretching	N-H stretch	C-H asymmetric stretch	Asymmetrical stretching in O=C=O	Stretching vibration in C-O	Carbonyl group	N-H bending	O-H bending	P=O stretch	C-N and C-O stretch	N-H symmetric deformation	Hetero-oxy compounds
64-200°C												
200-300°C			√	√	√	√	√	√	√		√	√
300-450°C	√	√	√	√	√	√	√	√	√	√	√	√
>450°C			√		√	√			√	√	√	

**Table:** (c) Roasted peanut functional group summary

Temperature (°C)	O-H stretching	C-H asymmetric stretch	Asymmetrical stretching in O=C=O	Stretching vibration in C-O	Carbonyl group	O-H bending	C-O stretch	P=O stretch	Hetero-oxy compounds
64-200°C	√		√		√				
200-300°C	√	√	√		√	√	√	√	√
300-450°C	√	√	√	√	√	√	√	√	√
>450°C		√	√	√	√				

**Table 3:** Summary of maximum degradation temperatures for *mopane* worms, sweet corn, and peanuts.

Parameter	Sweet corn	*Mopane* worms	Peanuts
*T*_5%_ (°C)	165	106	236
*T*_50%_ (°C)	384	398	419
*T*_max1_ (°C)	188	106	249
*T*_max2_ (°C)	248	403	322
*T*_max3_ (°C)	315	nd	453
*T*_max4_ (°C)	432	nd	nd
Char yield at 800°C (%)	20.4	21.94	15.08

**Table 4:** Heavy metal concentrations and health risk assessment values.

Element	Average concentration (mg kg^−1^) %RSD ≤ 17.8			EDI (mg kg^−1^ day^−1^)			Rf_*D*_ (mg kg^−1^ day^−1^)	Hazard quotient			CSF (kg-day mg^−1^)	Target cancer risk		
	Worm	Peanut	Sweet corn	Worm	Peanut	Sweet corn		Worm	Peanut	Sweet corn		Worm	Peanut	Sweet corn
As	22.5	18.2	74.6	0.0013	0.0074	0.261	0.003	0.424	2.45	86.9	1.5	0.0019	0.011	0.391
Cd	nd	nd	nd				0.025				6.3			
Co	nd	nd	nd				0.003							
Cr	nd	nd	nd				0.035				0.5			
Ni	nd	nd	nd				0.02							
Pb	nd	nd	nd				0.015				0.0085			

## Data Availability

Data is available on request.

## Additional Points

*Significance of the Study.* The TGA-FTIR was used to analyze the evolved gas products from roasted *mopane* worms, sweet corn, and peanuts in order understand the effect of heat treatment during roasting of the food products. The study showed that *mopane* worms had higher thermal stability and protein content compared to the sweet corn and peanuts. The evolution of NH and CN profiles during heating of *mopane* worms was attributed to the presence of amino acid proteins, and these were absent in the other food sources.
